# Perceptions of diagnosis and management of patients with acute respiratory distress syndrome: a survey of United Kingdom intensive care physicians

**DOI:** 10.1186/1471-2253-14-87

**Published:** 2014-10-02

**Authors:** Ahilanandan Dushianthan, Rebecca Cusack, Nigel Chee, John-Oliver Dunn, Michael PW Grocott

**Affiliations:** Department of Critical Care, Portsmouth Hospitals NHS Trust Queen Alexandra Hospital, Southwick Hill Road, Cosham, PO6 3LY UK; Critical Care Research and Anaesthesia Unit CE-93, MP-24, E-level, Centre Block, University Hospital Southampton NHS Foundation Trust, Southampton, SO16 6YD UK; Critical Care Research Area, Southampton NIHR Respiratory Biomedical Research Unit, University Hospital Southampton NHS Foundation Trust, Southampton, SO16 6YD UK; Integrative Physiology and Critical Illness Clinical and Experimental Sciences, Faculty of Medicine, University of Southampton, University Hospital Southampton NHS Foundation Trust, Southampton, SO16 6YD UK; Critical Care Unit, The Royal Bournemouth and Christchurch Hospitals NHS Foundation Trust, Castle Lane East, Bournemouth, BH7 7DW UK

**Keywords:** Acute respiratory distress syndrome, Hypoxia, Guidelines, Survey

## Abstract

**Background:**

Acute respiratory distress syndrome (ARDS) is a potentially devastating refractory hypoxemic illness with multi-organ involvement. Although several randomised controlled trials into ventilator and fluid management strategies have provided level 1 evidence to guide supportive therapy, there are few, established guidelines on how to manage patients with ARDS. In addition, and despite their continued use, pharmacotherapies for ARDS disease modulation have no proven benefit in improving mortality. Little is known however about the variability in diagnostic and treatment practices across the United Kingdom (UK). The aim of this survey, therefore, was to assess the use of diagnostic criteria and treatment strategies for ARDS in critical care units across the UK.

**Methods:**

The survey questionnaire was developed and internally piloted at University Hospital Southampton NHS Foundation Trust. Following ethical approval from University of Southampton Ethics and Research Committee, a link to an online survey engine (Survey Monkey) was then placed on the Intensive Care Society (UK) website. Fellows of The Intensive Care Society were subsequently personally approached via e-mail to encourage participation. The survey was conducted over a period of 3 months.

**Results:**

The survey received 191 responses from 125 critical care units, accounting for 11% of all registered intensive care physicians at The Intensive Care Society. The majority of the responses were from physicians managing general intensive care units (82%) and 34% of respondents preferred the American European Consensus Criteria for ARDS. There was a perceived decline in both incidence and mortality in ARDS. Primary ventilation strategies were based on ARDSnet protocols, though frequent deviations from ARDSnet positive end expiratory pressure (PEEP) recommendations (51%) were described. The majority of respondents set permissive blood gas targets (hypoxia (92%), hypercapnia (58%) and pH (90%)). The routine use of pharmacological agents is rare. Neuromuscular blockers and corticosteroids are considered occasionally and on a case-by-case basis. Routine (58%) or late (64%) tracheostomy was preferred to early tracheostomy insertion. Few centres offered routine follow-up or dedicated rehabilitation programmes following hospital discharge.

**Conclusions:**

There is substantial variation in the diagnostic and management strategies employed for patients with ARDS across the UK. National and/or international guidelines may help to improve standardisation in the management of ARDS.

**Electronic supplementary material:**

The online version of this article (doi:10.1186/1471-2253-14-87) contains supplementary material, which is available to authorized users.

## Background

Acute respiratory distress syndrome (ARDS) is a severe form of hypoxic respiratory failure associated with significant morbidity and mortality in critically-ill patients. Although the reported mortality from ARDS has declined in interventional studies over the past decades [1], observed death rates remain high in observational studies [2]. Survivors of ARDS experience varying degrees of pulmonary, physical, cognitive, emotional and psychological disability leading to a substantial and sustained disease burden [3]. Since its first description in 1967 [4], several attempts have been made to define a diagnostic criteria, that reliably identifies a cohort of patients with the corresponding pathological changes to aid both the diagnosis and management of ARDS, and in targeted research. Amongst many, Murray’s lung injury score (LIS), American European Consensus Conference (AECC) diagnostic criteria and more recently the Berlin definition of ARDS are the most commonly adopted definitions of ARDS [5–7].

Despite several refinements, current definitions are still hampered by lack of specificity as demonstrated by autopsy studies [8, 9]. ARDS is arguably a heterogeneous spectrum of conditions with variable aetiology, response to treatment and natural progression. Moreover, whilst evolution in supportive therapy may be contributing to the apparent improved outcome, lack of effective treatments remains a major on-going challenge. Consequently, there is a lack of coherent clinical guidance (by any of the critical care authoritative bodies), that incorporates all of the care bundles required to manage ARDS. Comparable guidelines do exist for conditions such as the sepsis syndromes, which incorporate strategies to minimise the development of ARDS [10].

This survey was conducted to identify current clinical practice in relation to diagnostic criteria and treatment of ARDS, as well as to explore perceptions about the epidemiology of this condition. Additionally, information regarding participation in clinical research and the use of data management resources to identify patients with ARDS was also explored.

## Methods

The survey questionnaire was developed using an online electronic survey engine (Survey Monkey) by two critical care physicians and a critical care research fellow (RC, MPW and AD). This was internally piloted among the general intensive care physicians at University Hospital Southampton NHS Foundation Trust. The questionnaire was subsequently modified and finalised based on the feedback received. Ethical approval was obtained from the University of Southampton Ethics and Research Committee (ERGO Number 1242). A link to the survey was posted on The Intensive Care Society (ICS), UK, website and advertised on three occasions in the monthly electronic ICS newsletter. In addition, all intensive care physicians registered with the ICS were approached individually via e-mail to participate. The survey was conducted for a period of three months from October 2012 to December 2012. No formal consent was required for the survey participation.

The questionnaire was aimed at intensive care physicians managing adult patients (more than 18 years of age) with ARDS across all general and specialist units in the UK. The survey questionnaire explored the following themes (Additional file 1).

1. ***Diagnosis:*** The physician’s preference and use of existing diagnostic definitions for ARDS.
2. ***Epidemiology:*** The physician’s perceptions on the epidemiology of ARDS within the UK.
3. ***Management:*** The ventilation, fluid balance, pharmacological strategies and rescue measures adopted in the management of patients with ARDS.
4. ***Post discharge rehabilitation:*** The availability of generic and specialist rehabilitation programmes.
5. ***Participation in clinical trials***
6. ***Data gathering:*** The availability of health informatics to identify patients with ARDS.

Numerical data are presented as percentages of total respondents to that particular question.

## Results

### Characteristics of respondents

**Figure 1:**
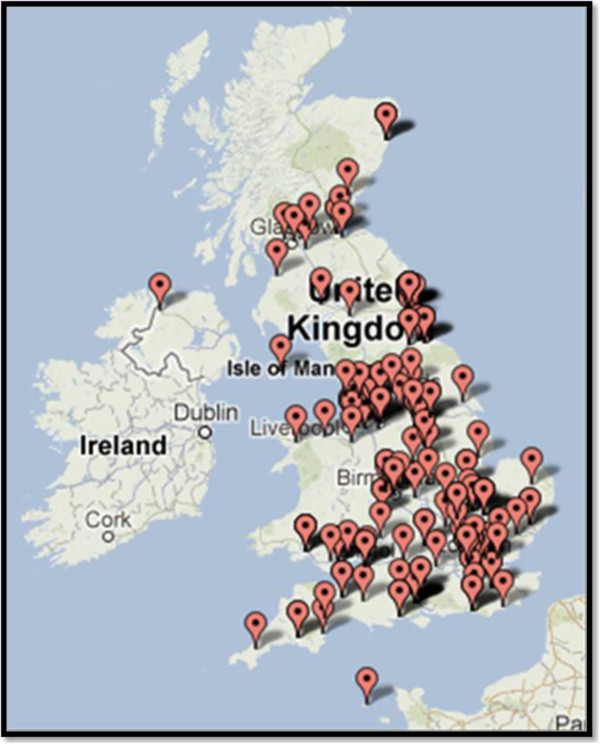
**Pictorial representation of the locations of hospitals from which the responses obtained.**

One hundred and ninety one respondents from 125 hospitals replied to the questionnaire (Figure 1). This was an 11% response rate from all critical care physicians registered at The Intensive Care Society, UK. Eighty two per cent of the respondents were from England, followed by Scotland (9%), Wales (6%), and Northern Ireland (0.5%). Three percent were from outside of the UK (Australia, Canada, Oman and the Channel Islands). Most of the respondents were physicians managing general intensive care units (82%) followed by specialist cardiac (8%), neuroscience (3%) and respiratory units (2%). The remainder comprised other specialist units including burns, hepatobiliary, transplant and mixed units. Each unit on average had 16 beds (range 4–106), accounting for 2.6% of total hospital beds. Ninety-seven per cent of respondents were consultants with the remainder consisting of senior trainees (3%). We did not further analyse responses by seniority of respondent or geographical location.

### Diagnostic definitions

**Figure 2:**
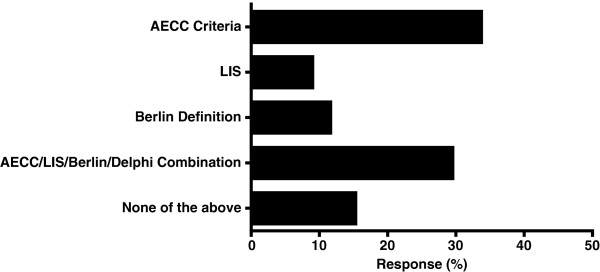
**Diagnostic definitions utilised to identify patients with ARDS.** Expressed as percentage of total responses. AECC, American European Consensus Criteria [6]; LIS, Murray’s Lung Injury Score [5]; Berlin, Berlin definition of ARDS [7]; Delphi, Delphi Consensus Criteria for ARDS [11].

All of the respondents answered this question and the most frequently used definition for ARDS was the AECC criteria. Thirty per cent used a combination of AECC/LIS/Berlin definition and Delphi Consensus Criteria [5–7, 11], while 15% used no rigid classifications. The Berlin definition of ARDS and Murray’s LIS was used alone to identify patients by 12% and 9% respectively (Figure 2). Although, AECC criteria was the commonest definition utilised, the use of a pulmonary artery catheter in this context was not further assessed by the questionnaire.

### Epidemiology

Ninety seven and 100% of respondents respectively replied to the questions regarding the incidence and mortality of ARDS. Forty five percent felt that the incidence of ARDS was declining and 63% felt that mortality was decreasing. Approximately one third felt that the incidence and mortality are static in their ARDS population (38% and 30% respectively). Less than 10% felt that incidence and mortality of ARDS was increasing. Opinions were evenly spread across the regions regardless of size and nature of the intensive care units or whether they were teaching or district general hospital units.

### Ventilation strategy

Ninety six percent responded to the question regarding primary ventilation strategies for ARDS. This question was primarily based on the ARDSnet protocol [12]. Thirty four per cent of respondents were fully compliant with ARDSnet protocol. An additional 51% were partially compliant with deviations in the PEEP recommendations noted. High frequency oscillatory ventilation (HFOV) was used by 13% as a primary ventilation strategy. These units were in-general based in university hospitals (70%). Airway pressure release ventilation (APRV), extracorporeal lung support (ECLS) and extracorporeal CO_2_ removal devices (ECCO_2_R) were only used by few respondents (<5%) as a primary ventilation strategy during the early stages of ARDS.

Respondents titrated positive end expiratory pressure (PEEP) according to different parameters. These included: the degree of hypoxia (74%), the ARDSnet protocol (34%) and the lower inflection point of the inspiratory pressure-volume curve (31%). The use of thoracic ultrasound, oesophageal pressures, end expiratory transpulmonary pressures and functional imaging such as electrical impedance tomography was rarely used to guide PEEP appliance (<2%). Computerised tomography (CT) scanning was only used in 3% of units to assess recruitability and all of these units were based in university hospitals.

For refractory hypoxemia/hypercapnia in the face of optimal ventilation, rescue measures included: recruitment manoeuvres (85%), prone positioning (84%), HFOV (50%), ECLS (33%) and ECCO_2_R devices (27%).

### Blood gas targets

**Figure 3:**
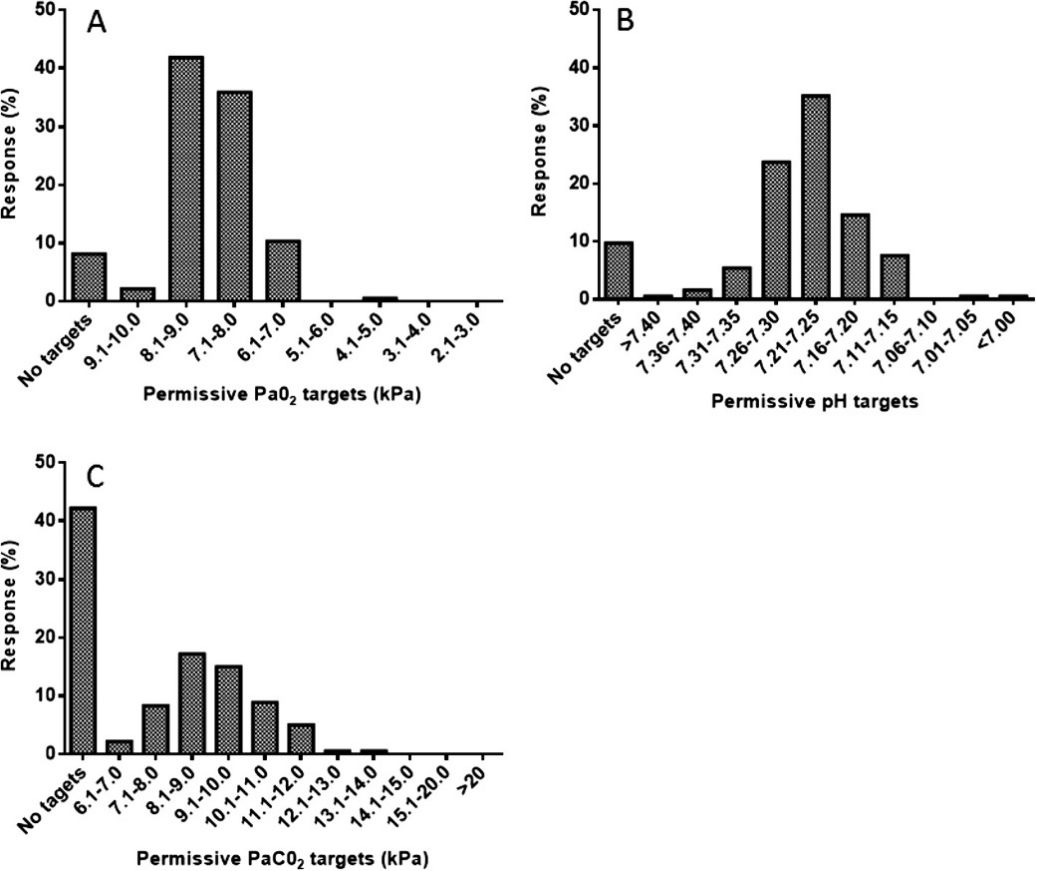
**Permissive arterial blood gas targets for Pa0**_**2**_ **(kPa) (A), pH (B) and PaC0**_**2**_ **(C).** Presented as percentage of total responses.

Almost all (>99%) provided answers for questions relating to blood gas targets. Forty two percent of respondents used no specific set limits for PaCO_2_ during mechanical ventilation, but of those who set limits, the majority (49%) were happy to maintain PaCO_2_ levels between 7–11 kilopascals (kPa) (Figure 3A). Although the level of permissive acidaemia varied amongst respondents, 35% aimed to maintain an arterial pH between 7.21-7.25 (Figure 3B). Permissive hypoxemia targets were set by the majority (91%), with 42% aiming for a PaO_2_ between 8.1-9.0 kPa, followed by 7.1-8.0 kPa in 36% (Figure 3C).

### Pharmacological treatments

We have categorised responses to the treatment frequency with available pharmacological agents as “routine”, “occasional”, “individualised according to patient” and “never”. For the purpose of this review, we have pooled together “occasional” and “individualised according to patient” groups.

#### β_2_-agonists

Ninety eight percent responded to this question, with 60% stating that they would use β_2_-agonists in the management of ARDS. Eleven per cent use β_2_-agonists routinely. We point out however, that the exact desired effect of β_2_-agonists was not clearly defined by the questionnaire (i.e. bronchodilatation or pulmonary oedema clearance).

#### Corticosteroids

Corticosteroids were used by 70% of the respondents, with only 6% using them routinely. The preferred corticosteroid was methylprednisolone (56%), followed by hydrocortisone (41%). Prednisolone use was scarce (3%). There was also considerable variation in the dose of corticosteroids prescribed (Table 1). Among those respondents who prescribed corticosteroids (70% of total), eighteen per cent initiated corticosteroid therapy less than 72 hours after the onset of ARDS, a further 12% within one week, 38% between 7 and 14 days and 15% after two weeks. The duration of therapy was ≤1 week in 56% and between 8–14 days in 34%. Corticosteroids are being used for their anti-fibrotic properties alone in 42% and anti-inflammatory effect alone in 23%. The remaining 25% suggested they are using corticosteroids for both clinical effects. The method of corticosteroid cessation was abrupt in 37% and by a tapered reducing-dose regime in 63%. Free text comments highlighted that the use of corticosteroids were dependent on several other factors. These included: concomitant use of vasopressors (especially increasing doses); the presence of sepsis; radiological (CT) evidence of ARDS with active lung fibrosis; treatment failure/non-progressing patients; and evidence of endogenous steroid deficiency.

**Table 1:** **The type and dose of steroid therapy initiated for acute respiratory distress syndrome**

Type of corticosteroid	Dose (mg/kg/day)					Total response (N)
	≤1 mg	2 mg	3 mg	4 mg	≥5 mg	
Hydrocortisone	23.6 (%)	33.3 (%)	31.4 (%)	7.8 (%)	3.9 (%)	51
Prednisolone	100 (%)	0 (%)	0 (%)	0 (%)	0 (%)	3
Methylprednisolone	42.0 (%)	27.5 (%)	1.5 (%)	5.8 (%)	23.2 (%)	69

The data expressed as a percentage of responses to the total response for the type of corticosteroid initiated.

#### Neuromuscular blocking agents (NMB)

Eighty three percent of respondents use “occasional” or “individualised NMB as part of the management of ARDS. Fifteen per cent of respondents describe using NMB “routinely”.

#### Prostaglandins or their derivatives

The majority of respondents (56%) reported never using prostaglandins or their derivatives in the management of ARDS, whilst 1.6% reported using them “routinely”. The remaining responses (42%) were equally divided between “occasional” and “individualised” use.

#### Statins

Most had “never” used statins (74%) and few used them “routinely” (3%). The remainder declared that statin use was “occasional” or “individualised” according to the patient (or indeed if part of a clinical research study).

#### Others

Most commented that they rarely used pulmonary surfactants (5%), heliox (7%) or nitric oxide (29%) as part of their pharmacological treatment strategy for patients with ARDS. Immunonutrition was used “routinely” by 9% and “occasional/individualised” according to patient requirement by 24%.

### Fluid balance

We asked specific daily fluid balance targets for patients with ARDS and this was answered by 98%. Thirty two percent (32%) would aim a negative balance of between 500–1000 millilitres per day (ml day^−1^), 23% aim for a negative balance of up to 500 ml day^−1^, 20% aim a neutral fluid balance and 9% would accept a positive balance up to 500 ml day^−1^. Additional comments suggested that the patient’s cardiovascular status and any evidence of renal impairment would dictate the target fluid balance.

### Tracheostomy

We asked whether tracheostomy is considered in this patient group routinely, occasionally or rarely, and further subdivided these categories into early (before 7 days) or late (after 7 days). This was answered by 98%. Most would perform a tracheostomy routinely (58%), with 64% stating that a late tracheostomy is the preferred option. Thirty four percent would perform tracheostomy occasionally (again the majority after 7 days). Tracheostomy was performed rarely by just 5%.

### Follow-up and the availability of rehabilitation programmes

**Figure 4:**
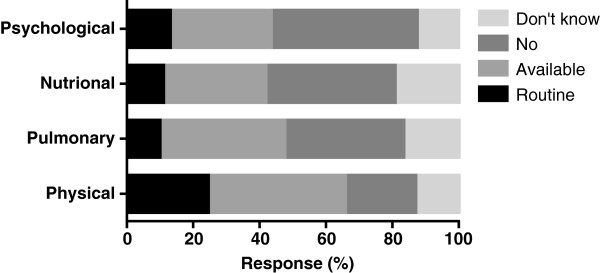
**Availability of specific rehabilitation programmes following discharge for ARDS patients.** Expressed as percentage of total responses.

Forty two percent (42%) follow-up this cohort of patients routinely after discharge. 98% responded to the question regarding the availability of any specific rehabilitation programmes following ICU discharge. Routine physical and pulmonary rehabilitation was available at 25% and 10% of respondent’s units respectively. Availability of routine nutritional and psychological support was not common (Figure 4).

### Participation into clinical research and data collection

Half of the respondents enrol their patients into clinical research. ARDS disease-specific data collection was performed by 25% and this was available electronically in about two thirds. Research participation resulted in specific ARDS data collection in a further 25%. No form of specific data collection was undertaken in 42% of the respondent’s units.

## Discussion

This is the first comprehensive survey describing diagnostic criteria, perceptions about epidemiology and treatment approaches for the management of ARDS in the UK. Despite significant morbidity and mortality from ARDS, there remains a lack of clarity with regards to the exact disease burden and a lack of consistency in management strategies adopted in the UK. Overall, we received 191 responses from 125 intensive care units of which 121 were within the UK. It must be highlighted that several major trials into ARDS were published around the time of this survey, which may make the responses to some questions difficult to interpret and the responses we gleaned may not now be a reflection of current clinical practice [13–15].

Although several diagnostic definitions have evolved over the years in an attempt to homogenise the heterogeneous ARDS population, the utility of these criteria outside clinical research remains controversial and their traction limited. Our survey conducted on intensive care physicians across the UK, indicates that significant variations remain in the preference as to which of the existing diagnostic definitions for ARDS is used. Despite the recent changes, most intensive care physician we surveyed still seem to prefer the AECC diagnostic criteria to the Berlin definition of ARDS. However, this survey was conducted shortly after the proposal of Berlin definition and the less frequent use of this definition compared to AECC criteria, may be as a result of lag time between publication and clinical implementation. That being said, 15% used no specific definitions for ARDS with most of those feeling that these definitions provide no additional benefit in routine clinical practice. Furthermore, free text comments described how some clinicians used clinical judgement alone (2%), whilst others felt that the use of definitions outside trials added no clinical value as long as lung protective ventilation strategies were adopted (3%). Indeed this raises a major issue and may have clinical implications in ARDS patient’s care and trial recruitment. If a specific target population is not identified using these definitions, it would be rather difficult to conceive treatment strategies translated from clinical trials or to test any novel therapy in that particular population.

Regarding the incidence and mortality of ARDS in the UK, there are no prospective large epidemiological studies to date. Webster conducted the first documented epidemiological study into ARDS across Yorkshire in 1988 (again a survey) [16]. Following this, Abel performed a single centre observational study between 1990 and 1997 [17]. This single centre study suggested that the mortality of ARDS had declined over this period from 66% to 44%. The ALIVE study (epidemiology and outcome of acute lung injury in European intensive care units) recruited patients from across Europe, also enrolling patients from the UK. Among this cohort the hospital mortality of ARDS was 58% [18]. Among the recent interventional randomised controlled trials into ARDS (BALTI-2 and OSCAR), the overall mortality was 29% and 41% respectively [13, 19]. This variation in reported mortality is likely to be due to several reasons including the intervention performed, inclusion and exclusion criteria, the population studied and a variable application of lung protective ventilation. Respondents to our survey felt that the incidence and mortality in ARDS is decreasing and this is consistent with clinical trials published by the ARDS network over the past decade [1]. A large multi-centre, prospective, observational study, examining the burden of acute hypoxic respiratory failure is currently complete and awaiting publication. This may provide additional morbidity and mortality data, which may complement the existing literature (LUNG SAFE, NCT02010073).

There were significant variations in the use of pharmacological agents for the management of patients with ARDS, although most of which were in keeping with the established evidence or the lack thereof. This particularly applied to the use of pulmonary surfactants, heliox and nitric oxide. Despite the lack of clinical evidence and even with the possibility of harm [13, 14], about 60% of respondents use β_2_-agonists as part of their treatment strategy, from routinely to individualised according to each patient. However, the specific reasons for their use (either for bronchodilatation or alveolar fluid clearance) and the mode of delivery (nebulised or intravenous) were not addressed by the questionnaire. It must be highlighted that the BALTI-2 study investigating the use of intravenous salbutamol in ARDS patients was published closer to the time of this survey and the perceived use of β_2_-agonists in our study may not be a reflection of current clinical practice [13]. The use of corticosteroids was much more common (>70%), although variation remains in the type, dose and duration of corticosteroids given and in the method of cessation. Most respondents preferred methylprednisolone at a dose of 0.5-1 mg kg^−1^, with treatment commencing between 7 and 14 days. Despite the lack of evidence and even with the possibility of harm, 15% of respondents still use corticosteroids after 14 days [20].

Protective lung ventilation with low tidal volumes is the primary ventilation strategy adopted by many since the publication of the seminal ARDSnet study [12]. However, it is not always possible to comply with this strategy of ventilation, as it can be associated with worsening of gas exchange [21]. Among the respondents, the primary ventilation strategy is principally based on ARDSnet protocol, though with frequent deviations to the recommended PEEP, tidal volumes and fraction of inspired oxygen (Fi0_2_). HFOV is utilised by 13% as a primary ventilation strategy, and 50% would use it as a rescue method if there was no improvement (or indeed worsening) in oxygenation/carbon dioxide clearance despite optimal ventilation. This survey was conducted prior to the recently published negative outcome studies into HFOV in ARDS [19, 22], so consequently the results from this survey may not be representative of current clinical practice.

Fluid balance is thought to be critical in the management of patients with ARDS. Studies have suggested that maintaining a neutral cumulative fluid balance is associated with an improved patient outcome [23]. Indeed the extent of a positive fluid balance in critically ill patients is associated with worsening clinical outcome [24]. In our survey, most respondents (>50%) preferred a negative overall cumulative fluid balance for their patients and this is in keeping with current evidence.

ARDS survivors are left with significant physical, cognitive and psychological sequelae. Little is known about the availability of routine rehabilitation programmes following discharge from UK hospitals. Only a quarter of respondents had facilities for routine physical rehabilitation following discharge and there remains a significant lack in nutritional and psychological support. This survey highlights the deficiency in specific rehabilitation programmes for improving patient morbidity following intensive care discharge and prospective epidemiological studies are needed to assess the precise health, social and economic implications of this lack in service provision.

The participation of 50% of our responders into ARDS-specific clinical research is encouraging. The continued lack of clinical benefit from randomised controlled trials into ARDS reiterates the importance of on-going research in this area. In the UK there are no data registries specific to ARDS, and the Intensive Care National Audit and Research Centre (ICNARC) case mix programme, which mainly concentrates on entry diagnosis, does not identify the majority of patients who develop ARDS during their intensive care admission. Research into any specific disease requires an accurate epidemiologic assessment for resource allocation and trial coordination. This lack of clarity in the true epidemiology of ARDS, not only for research purposes but also to recognise the nationwide impact of this deadly syndrome, is therefore ongoing.

Our survey has several limitations. Only 11% of total fellows registered on The Intensive Care Society, UK, responded to the questionnaire, which also included a small proportion trainees (3% overall). This low response rate may not be a true representation of the overall UK practice and highlights the limitation of this data on generalisability and external validity. Secondly, our method of electronic approach and participation may have resulted in some degree of selection bias as recruitment via the ICS website and e-mail list may have inadvertently selected a particular type of intensive care doctor. Furthermore, the respondents may have had a particular interest in ARDS management and thus our survey may not be an overall reflection of the generalised UK practice. This may possibly explain the high proportion of research participation among our responders. Thirdly, we cannot rule out sampling bias due to the nature of the recruitment of respondents. The sampling time for this survey was contemporaneous with several major UK randomised clinical trials into ARDS, that required high levels of engagement from UK-based critical care physicians (OSCAR, December 2007 - July 2012; BALTI-2, December 2006 - March 2010; TracMan, November 2004 - January 2011) [13, 19, 25]. Indeed, subsequent publication of the results from these trials makes the responses to some questions difficult to interpret.

## Conclusions

The survey examines the critical care physician’s views and practices in relation to ARDS management within the UK between October and December 2012. We have demonstrated considerable variation in both perceptions and practices. The use of some supportive therapies with a strong evidence base such as ventilator strategies are routinely used. Whereas, diagnostic criteria and pharmacotherapies are inconsistently applied. This degree of variation supports the need for the development of evidence based guidelines in this area.

### Key messages

-  The majority of clinicians use AECC or Berlin criteria to define ARDS.
-  Most clinicians use ARDSnet guidelines (at least to some degree) to manage their patients.
-  Advanced ventilation techniques are used by less than half the clinicians.
-  There is little standardisation in the use of pharmacotherapies.

## Electronic supplementary material

Additional file 1: **Survey questionnaire.** (PDF 264 KB)

## Abbreviations

AECC: American European Consensus Conference
ALIVE: Epidemiology and outcome of acute lung injury in European intensive care units
ALTA: The albuterol for the treatment of acute lung injury
APRV: Airway pressure release ventilation
ARDS: Acute respiratory Distress syndrome
BALTI-2: Effect of intravenous β_2_ agonist treatment on clinical outcomes in acute respiratory distress syndrome
ECCO2R: Extracorporeal CO_2_ removal system
ECLS: Extracorporeal lung support
HFOV: High frequency oscillatory ventilation
ICNARC: Intensive Care National Audit and Research Centre
ICS: Intensive Care Society
ICU: Intensive Care Unit
LIS: Lung injury score
NMB: Neuromuscular blocker
OSCAR: High-Frequency Oscillation for Acute Respiratory Distress Syndrome
OSCILLATE: High-Frequency Oscillation in Early Acute Respiratory Distress Syndrome
PEEP: Positive end-expiratory pressure
TracMan: Effect of early vs late tracheostomy placement on survival in patients receiving mechanical ventilation.

## References

[1] Spragg RG, Bernard GR, Checkley W, Curtis JR, Gajic O, Guyatt G, Hall J, Israel E, Jain M, Needham DM, Randolph AG, Rubenfeld GD, Schoenfeld D, Thompson BT, Ware LB, Young D, Harabin AL (2010). Beyond mortality: future clinical research in acute lung injury. Am J Respir Crit Care Med.

[2] Villar J, Blanco J, Añón JM, Santos-Bouza A, Blanch L, Ambrós A, Gandía F, Carriedo D, Mosteiro F, Basaldúa S, Fernández RL, Kacmarek RM, ALIEN Network (2011). The ALIEN study: incidence and outcome of acute respiratory distress syndrome in the era of lung protective ventilation. Intensive Care Med.

[3] Herridge MS, Tansey CM, Matté A, Tomlinson G, Diaz-Granados N, Cooper A, Guest CB, Mazer CD, Mehta S, Stewart TE, Kudlow P, Cook D, Slutsky AS, Cheung AM, Canadian Critical Care Trials Group (2011). Functional disability 5 years after acute respiratory distress syndrome. N Engl J Med.

[4] Ashbaugh DG, Bigelow DB, Petty TL, Levine BE (1967). Acute respiratory distress in adults. Lancet.

[5] Murray JF, Matthay MA, Luce JM, Flick MR (1988). An expanded definition of the adult respiratory distress syndrome. Am Rev Respir Dis.

[6] Bernard GR, Artigas A, Brigham KL, Carlet J, Falke K, Hudson L, Lamy M, Legall JR, Morris A, Spragg R (1994). The American-European Consensus Conference on ARDS. Definitions, mechanisms, relevant outcomes, and clinical trial coordination. Am J Respir Crit Care Med.

[7] Definition Task Force ARDS, Ranieri VM, Rubenfeld GD, Thompson BT, Ferguson ND, Caldwell E, Fan E, Camporota L, Slutsky AS (2012). Acute respiratory distress syndrome: the Berlin definition. JAMA.

[8] Ferguson ND, Frutos-Vivar F, Esteban A, Fernández-Segoviano P, Aramburu JA, Nájera L, Stewart TE (2005). Acute respiratory distress syndrome: under recognition by clinicians and diagnostic accuracy of three clinical definitions. Crit Care Med.

[9] Thompson BT, Matthay MA (2013). The Berlin definition of ARDS versus pathological evidence of diffuse alveolar damage. Am J Respir Crit Care Med.

[10] Dellinger RP, Levy MM, Rhodes A, Annane D, Gerlach H, Opal SM, Sevransky JE, Sprung CL, Douglas IS, Jaeschke R, Osborn TM, Nunnally ME, Townsend SR, Reinhart K, Kleinpell RM, Angus DC, Deutschman CS, Machado FR, Rubenfeld GD, Webb SA, Beale RJ, Vincent JL, Moreno R, Surviving Sepsis Campaign Guidelines Committee including the Pediatric Subgroup (2013). Surviving sepsis campaign: international guidelines for management of severe sepsis and septic shock: 2012. Crit Care Med.

[11] Ferguson ND, Davis AM, Slutsky AS, Stewart TE (2005). Development of a clinical definition for acute respiratory distress syndrome using the Delphi technique. J Crit Care.

[12] The Acute Respiratory Distress Syndrome Network (2000). Ventilation with lower tidal volumes as compared with traditional tidal volumes for acute lung injury and the acute respiratory distress syndrome. N Engl J Med.

[13] Gao Smith F, Perkins GD, Gates S, Young D, McAuley DF, Tunnicliffe W, Khan Z, Lamb SE, BALTI-2 study investigators (2012). Effect of intravenous beta-2 agonist treatment on clinical outcomes in acute respiratory distress syndrome (BALTI-2): a multicentre, randomised controlled trial. Lancet.

[14] Matthay MA, Brower RG, Carson S, Douglas IS, Eisner M, Hite D, Holets S, Kallet RH, Liu KD, MacIntyre N, Moss M, Schoenfeld D, Steingrub J, Thompson BT, National Heart, Lung, and Blood Institute Acute Respiratory Distress Syndrome (ARDS) Clinical Trials Network (2011). Randomized, placebo-controlled clinical trial of an aerosolized beta (2)-agonist for treatment of acute lung injury. Am J Respir Crit Care Med.

[15] Papazian L, Forel JM, Gacouin A, Penot-Ragon C, Perrin G, Loundou A, Jaber S, Arnal JM, Perez D, Seghboyan JM, Constantin JM, Courant P, Lefrant JY, Guérin C, Prat G, Morange S, Roch A, ACURASYS Study Investigators (2010). Neuromuscular blockers in early acute respiratory distress syndrome. N Engl J Med.

[16] Webster NR, Cohen AT, Nunn JF (1988). Adult respiratory distress syndrome–how many cases in the UK?. Anaesthesia.

[17] Abel SJ, Finney SJ, Brett SJ, Keogh BF, Morgan CJ, Evans TW (1998). Reduced mortality in association with the acute respiratory distress syndrome (ARDS). Thorax.

[18] Brun-Buisson C, Minelli C, Bertolini G, Brazzi L, Pimentel J, Lewandowski K, Bion J, Romand JA, Villar J, Thorsteinsson A, Damas P, Armaganidis A, Lemaire F, ALIVE Study Group (2004). Epidemiology and outcome of acute lung injury in European intensive care units. Results from the ALIVE study. Intensive Care Med.

[19] Young D, Lamb SE, Shah S, MacKenzie I, Tunnicliffe W, Lall R, Rowan K, Cuthbertson BH, OSCAR Study Group (2013). High-frequency oscillation for acute respiratory distress syndrome. N Engl J Med.

[20] Steinberg KP, Hudson LD, Goodman RB, Hough CL, Lanken PN, Hyzy R, Thompson BT, Ancukiewicz M, National Heart, Lung, and Blood Institute Acute Respiratory Distress Syndrome (ARDS) Clinical Trials Network (2006). Efficacy and safety of corticosteroids for persistent acute respiratory distress syndrome. N Engl J Med.

[21] Feihl F, Eckert P, Brimioulle S, Jacobs O, Schaller MD, Melot C, Naeije R (2000). Permissive hypercapnia impairs pulmonary gas exchange in the acute respiratory distress syndrome. Am J Respir Crit Care Med.

[22] Ferguson ND, Cook DJ, Guyatt GH, Mehta S, Hand L, Austin P, Zhou Q, Matte A, Walter SD, Lamontagne F, Granton JT, Arabi YM, Arroliga AC, Stewart TE, Slutsky AS, Meade MO, OSCILLATE Trial Investigators; Canadian Critical Care Trials Group (2013). High-frequency oscillation in early acute respiratory distress syndrome. N Engl J Med.

[23] Wiedemann HP, Wheeler AP, Bernard GR, Thompson BT, Hayden D, de Boisblanc B, Connors AF, Hite RD, Harabin AL, National Heart, Lung, and Blood Institute Acute Respiratory Distress Syndrome (ARDS) Clinical Trials Network (2006). Comparison of two fluid-management strategies in acute lung injury. N Engl J Med.

[24] Boyd JH, Forbes J, Nakada TA, Walley KR, Russell JA (2011). Fluid resuscitation in septic shock: a positive fluid balance and elevated central venous pressure are associated with increased mortality. Crit Care Med.

[25] Young D, Harrison DA, Cuthbertson BH, Rowan K (2013). Effect of early vs late tracheostomy placement on survival in patients receiving mechanical ventilation: the TracMan randomized trial. JAMA.

[CR26] The pre-publication history for this paper can be accessed here:http://www.biomedcentral.com/1471-2253/14/87/prepub

