# Supplementary material for: Perceptions of diagnosis and management of patients with acute respiratory distress syndrome: a survey of United Kingdom intensive care physicians
Source: BMC Anesthesiol. 2014 Oct 2;14:87. doi: 10.1186/1471-2253-14-87 (PMC4192350; doi:10.1186/1471-2253-14-87)
Supplement: Supplementary file 1 — Additional file 1: Survey questionnaire. (PDF 264 KB) [file 12871_2014_300_MOESM1_ESM.pdf]

*1. Your Hospital Name

*2. Is your ICU a specialist or general unit?

Type of Unit

Specify if other specialist unit

*3. How many ICU beds you have?
How many hospital beds you have?

ICU

Hospital

4. What diagnostic definitions do you use to identify patients with Acute Lung Injury (ALI)/ Acute Respiratory Distress Syndrome (ARDS)

- [ ] American European Consensus Conference Criteria
- [ ] Lung Injury Score
- [ ] Delphi Consensus Criteria
- [ ] Berlin definition of ARDS
- [ ] All of the above
- [ ] None of the above
- [ ] Other

Other (please specify)

5. In your opinion, the incidence of ARDS/ALI is;

- ○ Increasing
- ○ Decreasing
- ○ Static
- ○ Not sure

Comment

6. In your opinion, the mortality of ARDS/ALI is;

- Increasing
- Decreasing
- Static
- Not sure

Comment

7. Do you use the following pharmacological agents to treat patients with ALI/ARDS?

	Routinely	Occasionally	Individualised according to patient	Never
Beta-2 agonists (IV or nebulised)				
Corticosteroids				
Heliox				
Immunonutrition				
Neuromuscular agents				
Nitric oxide				
Prostaglandins or their derivatives				
Statins				
Surfactants				

Other (please specify)

8. If you have answered yes to the use of corticosteroids (Q8), what is the steroid preference, dose and duration of therapy

	Dose	Duration	Method of steroid cessation
Hydrocotisone			
Prednisolone			
Methylprednisolone			

Other (please specify dose and duration)

9. With regards to corticosteroids in ARDS/ALI, when do you initiate steroids and what is the reason for the initiation?

	How long after diagnosis of ARDS do you initiate?	Reason for initiation
Corticosteroids in ALI/ARDS		

Other (please specify)

10. What is your fluid balace strategy in patients with moderate to severe hypoxia related to ALI/ARDS

	Daily fluid balance targets	Preferred resuscitation fluid	If you aim negative balace, how do you achieve it?
Aims of fluid balance			

Comment

11. What is your primary ventilation strategy?

- ☐ Full compliance with ARDSNet protocol
- ☐ Partial compliance with ARDSNet protocol with deviation from PEEP recommendations
- ☐ Partial compliance with ARDSNet protocol with deviation from Tidal volume recommendations
- ☐ Partial compliance with ARDSNet protocol with deviation from Fi02 recommendations
- ☐ Does not use ARDSNet protocol
- ☐ High frequency oscillatory ventilation (HFOV)

Other (please specify)

12. What is your guidence for titration of PEEP?

- ☐ ARDSNet Protocol
- ☐ Degree of hypoxia
- ☐ Lower Inflection point of the inspiratory pressure-volume curve
- ☐ Plateau pressure
- ☐ Peak airways pressure
- ☐ Oesophageal pressure
- ☐ End-expiratory transpulmonary pressure
- ☐ Recruitability as assessed by chest ultrasound
- ☐ Recruitability as assessed by CT scan
- ☐ Functional imaging (Eg: electrical impedence tomography)

Other (please specify)

13. What are your permissive levels for hypercapnia, pH and hypoxia?

	Levels of PaC02	Levels of pH	Levels of Pa02
Permissive targets			

Other/comment

14. What are your rescue measures if no improvement despite maximal ventilation?

- [ ] Recruitment maneuvers
- [ ] Proning
- [ ] High Frequency Oscillatory Ventialtion (HFOV)
- [ ] CO_2 removal devices
- [ ] Extra-Corproreal Membrane Oxygenation (ECMO)

Other (please specify)

15. Do you consider trachestomy for ALI/ARDS patients?

Comment

16. Do you routinely follow-up these patients after discharge from hospital

Comment

17. Are the following rehabilitation programmes available after discharge?

	Routine	Available	No	Don't know
Physical rehabilitation	○	○	○	○
Pulmonary rehabilitation	○	○	○	○
Nutritional therapy	○	○	○	○
Psychological assessment and support	○	○	○	○

Comment

18. Do you enroll your patients with ALI/ARDS into clinical research?

- ○ Yes
- ○ No
- ○ Not sure

19. What data management system do you have to audit patients who have had ARDS/ALI?

- ☐ Pre-existing specific disease based (ARDS/ALI) data collection- electronic
- ☐ Pre-existing specific disease based (ARDS/ALI) data collection- manual
- ☐ Pre-existing data collection from research participation- electronic
- ☐ Pre-existing data collection from research participation- manual
- ☐ None
- ☐ Not sure

Other (please specify)
